# Evaluation of laboratory parameters and symptoms after parathyroidectomy in dialysis patients with secondary hyperparathyroidism

**DOI:** 10.1080/0886022X.2019.1666724

**Published:** 2019-10-01

**Authors:** Yi Zhang, Ying Lu, Sheng Feng, Zhoubing Zhan, Huaying Shen

**Affiliations:** Department of Nephrology, The Second Affiliated Hospital of Soochow University, Suzhou, China

**Keywords:** Parathyroidectomy, secondary hyperparathyroidism, iPTH, dialysis patients

## Abstract

**Objectives:** The aim of the study was to evaluate the laboratory parameters and symptoms after parathyroidectomy (PTX) in dialysis patients with secondary hyperparathyroidism (SHPT), and to briefly analyze the different therapeutic effects of the three surgical methods.

**Methods:** A total of 182 dialysis patients who underwent PTX between February 2012 and January 2018 at the Second Affiliated Hospital of Soochow University were included in this study and followed for 12 months. Laboratory parameters such as calcium (Ca), phosphorus (P), alkaline phosphatase (ALP), and intact parathyroid hormone (iPTH) were measured before and after operation. According to the follow-up time and type of operation, we calculated the percentage of laboratory indicators reaching the recommended range of the KDIGO guidelines after surgery. We also analyzed the improvement of bone pain and pruritus, as well as surgical complications.

**Results:** After the operation, the levels of iPTH, Ca, and P decreased significantly at each time point. ALP increased at the first postoperative week and gradually decreased to normal range after 3 months. Symptoms, such as bone pain and pruritus, were significantly relieved. According to the follow-up time and three surgical methods (subtotal parathyroidectomy, total parathyroidectomy, total parathyroidectomy plus autologous transplantation), we found that the ratio of each laboratory parameter reaching the recommended range of KDIGO guidelines was significantly different.

**Conclusion:** PTX is a safe and effective therapy for treating SHPT that is refractory to medical therapies and accompanied by related signs and symptoms in dialysis patients. All three operative techniques were effective in controlling SHPT.

## 1. Introduction

Secondary hyperparathyroidism (SHPT) is a major complication of dialysis patients. It is characterized by disorders of calcium and phosphorus metabolism, vascular calcification, and unbalanced bone metabolism [1]. Severe SHPT also causes bone pain and itching, thus contributing to poor quality of life in dialysis patients [2–5]. Studies have shown that the incidence of cardiovascular events and all-cause mortality in patients with chronic kidney disease with secondary hyperparathyroidism are significantly increased [6].

The treatment for SHPT includes non-calcium-containing phosphate binders, vitamin D sterols, vitamin D analogs, and calcimimetics [7]. However, these treatments do not always provide adequate control of SHPT, particularly among patients with severe parathyroid hyperplasia [8,9]. Successful parathyroidectomy can drastically decrease PTH levels, improve control of serum calcium and phosphorus levels, ameliorate symptoms related to SHPT, and reduce cardiovascular events and overall mortality in dialysis patients [10–12]. PTX is, thus, currently recommended in dialysis patients with severe SHPT who fail to respond to medical treatment [13]. Parathyroidectomy can be divided into three types: subtotal parathyroidectomy (S-PTX), total parathyroidectomy (T-PTX), and total parathyroidectomy plus autologous transplantation (PTX + AT) [14]. Nowadays, the optimal surgical treatment for secondary hyperparathyroidism is not well defined [15].

According to KDIGO guidelines [16,17], calcium is to be maintained within the normal range, as is phosphate. iPTH is to be maintained between two and nine times the upper limit of normal (130–600 pg/mL). In this study, we analyzed the trend of laboratory indicators and their ratio reaching the recommended range of KDIGO guidelines in terms of follow-up time and type of surgery. Meanwhile, the improvement of bone pain and pruritus was also counted.

## 2. Materials and methods

### 2.1. Inclusion criteria

Inclusion criteria included (1) patients aged >18 years and <70 years with SHPT with surgery indication and (2) dialysis patients with chronic kidney disease.

### 2.2. Exclusion criteria

Exclusion criteria included patients over 70 years of age, patients with severe surgical contraindications, patients with a history of liver dysfunction or hepatitis, patients with primary hyperparathyroidism, and patients receiving renal transplantation during the study period.

### 2.3. Surgery indications

Surgical indications included (1) intact parathyroid hormone (iPTH) level > 800 pg/mL complicated with hypercalcemia or hyperphosphatemia; (2) failure of conservative medical treatment and/or ineffectiveness of intensive dialysis; (3) complicated SHPT with severe clinical symptoms, such as bone and joint pain or pruritus; and (4) imaging examination by ultrasonography or emission computed tomography showing that at least one parathyroid gland was enlarged, with a diameter greater than 1 cm or a volume larger than 0.5 cm³, and had abundant blood flow signals. Exclusion criteria were as follows: (1) evidence of severe cardiopulmonary dysfunction, acute infection, abnormal liver function and coagulation index, or severe thyroid dysfunction; (2) patients who were unable to cooperate or had mental disorders; and (3) history of multiple neck surgeries.

### 2.4. Surgical technique

All the operations were performed by the chief surgeon of thyroid and breast surgery in our hospital, and two other surgeons worked together as assistants. The mode of operation was determined by the surgeon’s clinical experience. The amount of glands retained during subtotal parathyroidectomy was determined by the result of intraoperative exploration. The general principle is to retain more glands when they are small and less when they are large. If the diameter of glands is less than 1 cm, 1/2 of them will be retained, and if the diameter of glands is greater than 2 cm, 1/4–1/3 of them will be retained. For total parathyroidectomy with autotransplant, the most normal-appearing non-nodular areas of the parathyroid gland were divided into pieces of 1 mm^3^ in size after all parathyroid glands were removed. About 40–50 mg of parathyroid tissue was transplanted into forearm muscles without an arteriovenous fistula for hemodialysis. Intraoperative iPTH was collected within 10 min after gland excision.

### 2.5. Data collection

The serum iPTH, calcium, inorganic phosphate, and alkaline phosphatase levels were measured before the operation (Preop), during the operation (Intrao), and after the PTX at 1 week (1 w), 3 months (3 m), 6 months (6 m), and 12 months (12 m). The data of clinical symptoms (bone pain and pruritus), operative complications, and recurrence of SHPT were analyzed.

Serum calcium (NV 2.0–2.7 mmol/L), serum phosphorus (NV 0.8 1–1.55 mmol/L), and alkaline phosphatase (NV 4 5–135 mmol/L) were determined with an AU5000 automated chemistry analyzer (Olympus, Tokyo, Japan). Serum iPTH levels (NV12–88 pg/mL) were measured in an immunoradiometric assay (Nichols Institute Diagnostics, San Juan Capistrano, CA, USA). Compared with preoperative, intraoperative monitoring of iPTH resulting in a rate of decline > 70% was the criterion for a successful operation [18]. Diagnostic criteria for SHPT recurrence were iPTH > 300 pg/mL and symptoms, such as bone pain and pruritus, 6 months after the operation [19]. Persistent SHPT was defined as a threefold higher level of serum parathyroid hormone than the upper limit of the reference value on the third day after operation (the normal reference range of our hospital is 12–88pg/mL) [20]. Hypoparathyroidism was defined as the iPTH continued to be lower than 10 pg/mL after one year follow-up [21].

### 2.6. Statistical analysis

Data management and analysis were performed using IBM^®^ SPSS^®^ Statistics 22 for Windows^®^ software (IBM Corporation, New Orchard Road Armonk, New York, USA). All continuous variables were expressed as the mean ± standard deviation, and categorical variables were expressed as frequency (percentage). Continuous data were analyzed by the t-test, categorical data were analyzed with the Chi-square test, and skewed data were analyzed with variance. The measurement of data with skewed distributions were expressed by median (interquartile range), and a nonparametric rank sum test was used for inter-group comparison.

## 3. Results

### 3.1. Baseline characteristics

A total of 182 maintenance dialysis patients who underwent parathyroidectomy for severe forms of SHPT in our hospital from February 2012 to January 2018 were selected. There were 136 cases of hemodialysis, 43 cases of peritoneal dialysis, and 3 cases that converted from peritoneal dialysis to hemodialysis. Their baseline characteristics are summarized in Table 1.

**Table 1.** Baseline characteristics of 182 patients.

	Number of cases (value)	Scope or proportion
Age (years)	48.5 ± 10.8	18–70
Gender (male/female)	100/82	54.9%/45.1%
Dialysis durations (months)	97.0 ± 42.1	12–276
Primary disease		
Chronic glomerulonephritis	97	53.3%
Nephropathy of uncertain diagnosis	5	2.7%
Hypertensive nephropathy	33	18.1%
Nephropathy in hypertension	4	2.2%
Diabetic nephropathy	25	13.7%
Nephropathy in diabetes	7	3.8%
Anaphylatic purpura nephritis	3	1.6%
Lupus nephritis	8	4.4%
Clinical symptoms		
Bone pain	178	97.8%
Skin pruritus	176	96.7%
Operative method		
S-PTX	34	18.7%
T-PTX	28	15.4%
PTX + AT	120	65.9%

S-PTX: subtotal parathyroidectomy; T-PTX: total parathyroidectomy; PTX + AT: total parathyroidectomy plus autologous transplantation. Continuous variables were expressed as the mean ± standard deviation and categorical variables were expressed as frequency (percentage).

### 3.2. Surgical results

The number of parathyroid glands removed ranged from 1 to 5, with an average of 3.63. According to the statistics of the number of parathyroid glands excised during the operation, we know that 140 patients have excised 4 parathyroid glands, 9 patients have excised 1 parathyroid gland, 10 patients have excised 2 parathyroid glands, 22 patients have excised 3 parathyroid glands, and 1 patient has excised 5 parathyroid glands. Among the patients who underwent total parathyroidectomy, ectopic parathyroid (the fifth parathyroid) was found in one case, and was removed by thoracic surgery.

Pathologically, 158 (86.8%) patients had nodular hyperplasia, 19 (10.2%) had parathyroid adenoma, 5 (2.7%) had thyroid cancer, and underwent partial or total thyroidectomy. The success rate of PTX was 85.2% (155/182), 12.1% (22/182) patients had persistent SHPT, and 5.5% cases (8/146) resulted in SHPT recurrence 6 months after operation. Among the recurrent cases, five cases were treated with S-PTX and three cases were treated with PTX + AT. In detail, five cases were caused by residual gland recurrence and three cases by autograft recurrence. We found that among the recurrent patients, three cases chose reoperation, five cases chose drug conservative treatment. But this is different in persistent SHPT. Among them, 18 patients opted for reoperation, only two patients opted for medication, and another two patients lost to follow-up. One patient died of acute coronary syndrome during the perioperative period. The main problem after the operation was hypocalcemia, for which the incidence was 62.1%. A total of 101 patients were treated with intravenous calcium supplementation after operation, including 16 patients in S-PTX group, 24 patients in T-PTX group, and 67 patients in PTX + AT group. After treatment with various calcium supplementation schemes during hospitalization, the calcium level of all patients was improved steadily. According to statistics, the average dose of calcium supplementation after operation is about 2 g/day. Six months after operation, a small number of patients had elevated serum phosphorus. Our center used lanthanum carbonate and sevelamer for treatment. The average dose was 1 g/day and 2.4 g/day, respectively. Consistent with the statistical probability of complications after operation, there were two cases of transitory recurrent laryngeal nerve injury, which showed hoarseness but no dysphagia or dyspnea and improved after nutritional nerve treatment. One case of incision hemorrhage was improved after adequate drainage and hemostasis symptomatic treatment. Three patients had hypoparathyroidism after surgery, but fortunately, they did not have severe convulsions, limb numbness, gastrointestinal discomfort, and other adverse reactions. Eight cases of combined infection were improved after treatment and discharged. By the end of the study, 36 patients lost in follow-up. Among them, 6 patients underwent S-PTX, 4 patients underwent T-PTX, and 26 patients underwent PTX + AT, as shown in Table 2.

**Table 2.** General information on three surgical procedures (*n*).

	S-PTX	T-PTX	PTX + AT
Total	34	28	120
Recurrence	5	0	3
Persistent SHPT	4	2	16
Hypocalcemia	17	26	70
Intravenous calcium supplementation	16	24	67
Lost to follow-up	6	4	26

S-PTX: subtotal parathyroidectomy; T-PTX: total parathyroidectomy; PTX + AT: total parathyroidectomy plus autologous transplantation.

### 3.3. Differences in laboratory indicators and rate of reaching KDIGO recommended range (according to the follow-up time)

Compared with preoperative values, iPTH decreased significantly during and after the operation (1 week, 3 months, 6 months, and 12 months after operation) (*p* < 0.05). ALP increased at the first postoperative week and gradually decreased to the normal range after 3–12 months (*p* < 0.05). Serum calcium and phosphorus levels were also significantly decreased 1 week, 3 months, 6 months, and 12 months after operation (*p* < 0.05), as shown in Table 3.

**Table 3.** Comparison of differences in parameters before and after PTX.

	iPTH (pg/mL)	ALP (u/L)	Ca (mmol/L)	P (mmol/L)	Ca × P (mg²/dL²)
Preop (*n* = 182)	1638.75 (1182.10, 2330.68)	305.50 (163.00, 607.00)	2.73 ± 0.29	2.25 ± 0.50	75.88 ± 18.49
Intrao (*n* = 182)	204.20 (122.58, 367.08)^a^	/	/	/	/
1.w (*n* = 182)	30.75 (7.65, 108.73)^a^	321.70 (193.75, 653.75)^a^	1.95 ± 0.22^a^	1.16 ± 0.35^a^	27.98 ± 9.71^a^
3.m (*n* = 157)	54.30 (16.10, 214.75)^a^	223.00 (102.00, 443.00)^a^	2.00 ± 0.22^a^	1.22 ± 0.34^a^	30.26 ± 9.84^a^
6.m (*n* = 146)	63.65 (29.48, 244.50)^a^	107.50 (66.75, 216.50)^a^	2.07 ± 0.21^a^	1.27 ± 0.34^a^	32.80 ± 10.00^a^
12.m (*n* = 146)	59.20 (31.10, 166.43)^a^	99.00 (71.00, 135.00)^a^	2.26 ± 0.30^a^	1.49 ± 0.33^a^	42.10 ± 11.77^a^

PTX: parathyroidectomy; iPTH: intact parathyroid hormone; ALP: alkaline phosphatase; Ca: serum calcium; P: serum phosphorus; Ca × P: calcium phosphorus product; Preop: Preoperative; Intrao: intraoperative; 1.w: 1 week after operation; 3.m: 3 months after operation; 6.m: 6 months after operation; 12.m: 12 months after operation; /: not monitored. iPTH and ALP levels are expressed as the median (interquartile range); Ca, P, and Ca × P are expressed as the mean ± standard deviation; Continuous data were analyzed by the t-test. The measurement of data with skewed distributions were expressed by median (interquartile range), and a nonparametric rank sum test was used for inter-group comparison. A probability value of ^a^*p* < 0.05 was considered to be statistically significant.

**Figure 1.**
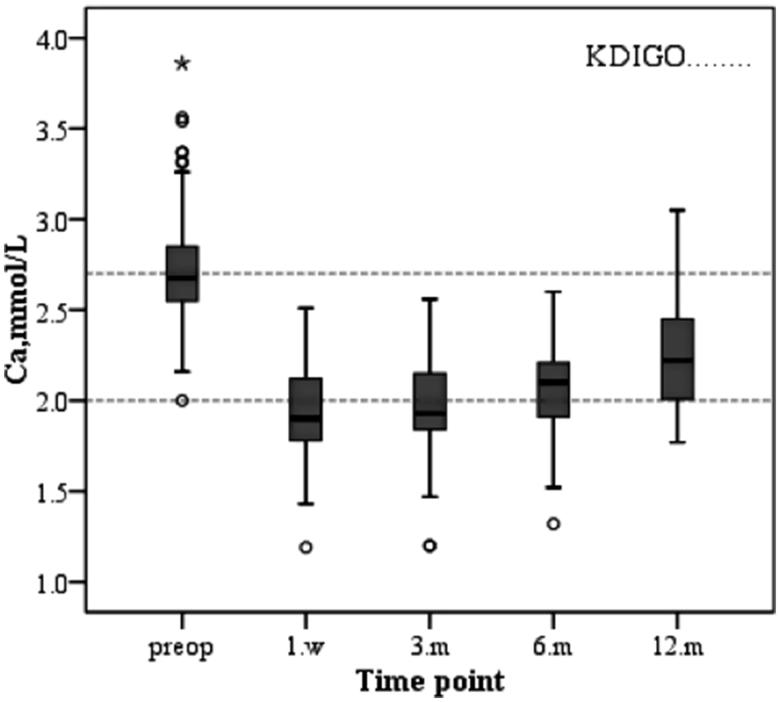
Changes in serum levels of calcium during the study period.

**Figure 2.**
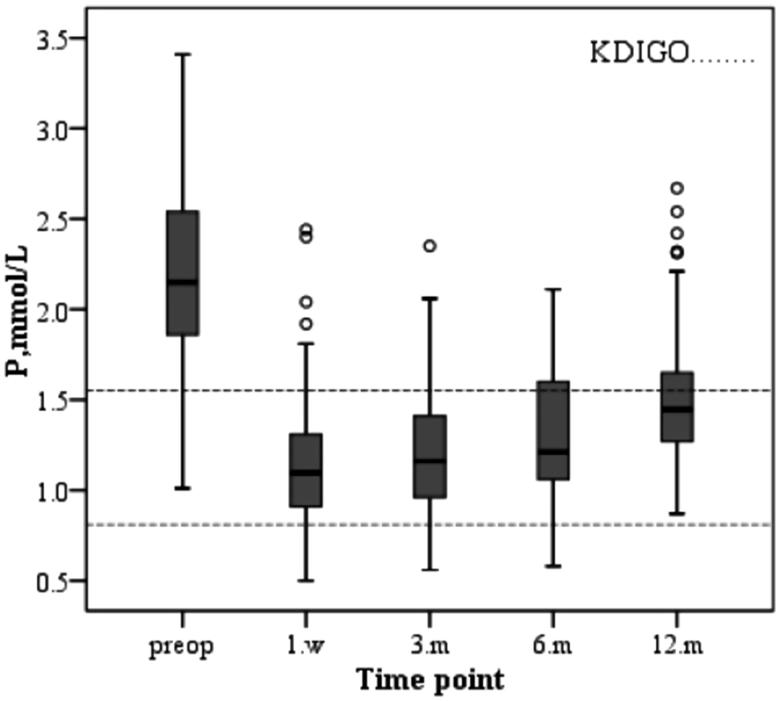
Changes in serum levels of phosphorus during the study period.

**Figure 3.**
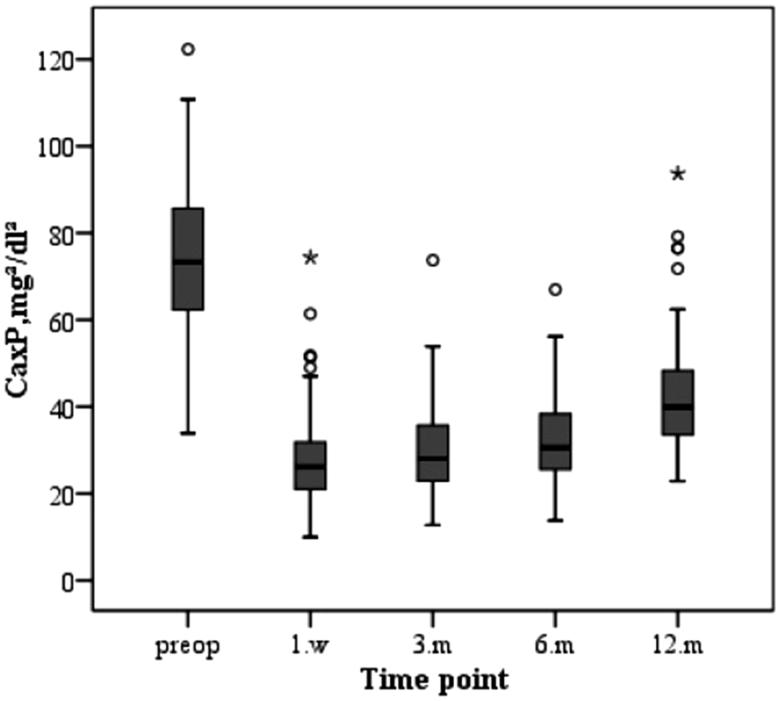
Changes in the Ca × P during the study period.

**Figure 4.**
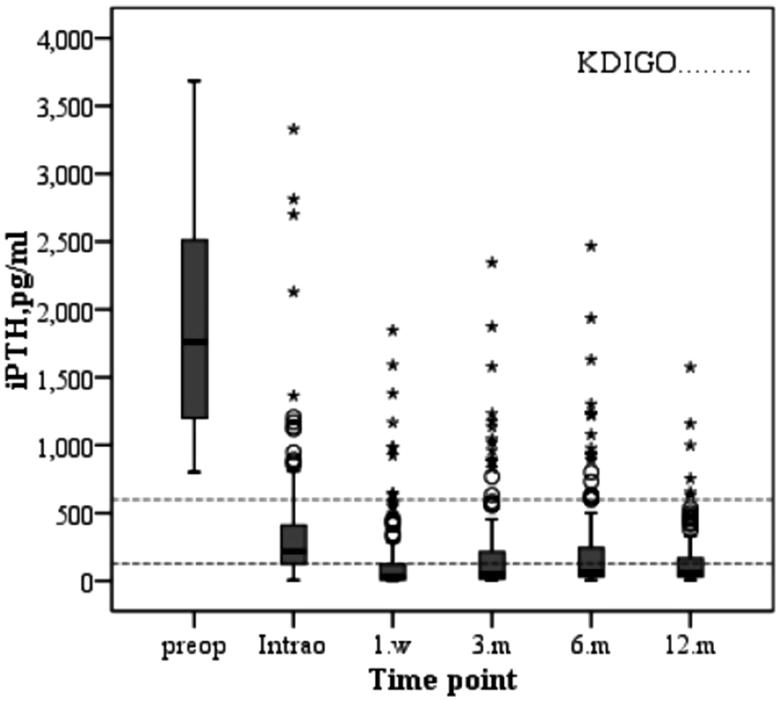
Changes in serum levels of iPTH during the study period.

**Figure 5.**
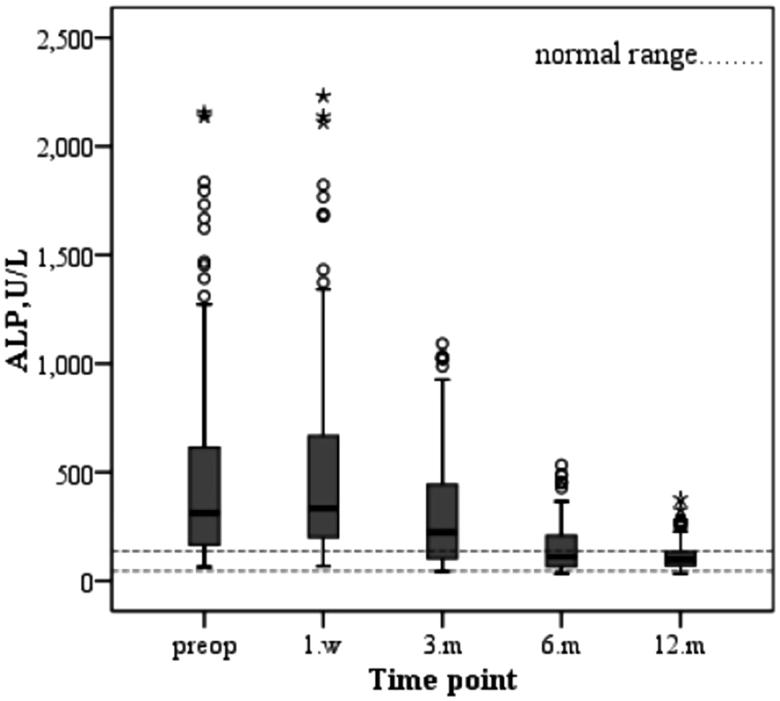
Changes in serum levels of ALP during the study period.

The changes in serum biochemical parameters and iPTH after parathyroidectomy are as follows. The serum calcium level of the patients decreased immediately after operation and increased slowly during the first year after surgery due to supplementation of calcium and active vitamin D3 (Figure 1). The varied trend of serum phosphorus levels was similar to that of serum calcium, both of which decreased after operation and increased slowly after operation and were stably maintained stability (Figure 2). Similarly, the change in the product of calcium and phosphorus is shown in Figure 3. The majority of patients who underwent parathyroidectomy failed to achieve the KDIGO iPTH target (Figure 4). The changing trend of ALP is shown in Figure 5.

**Figure 6.**
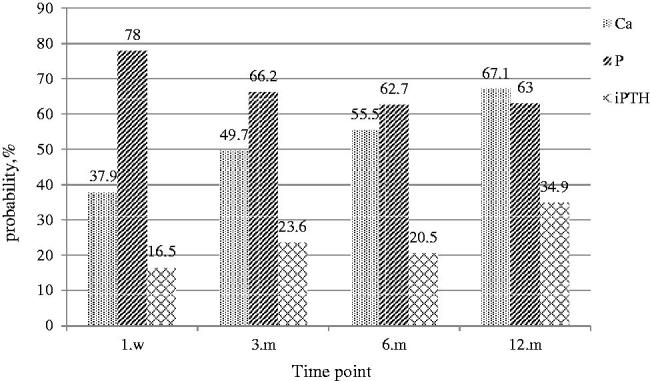
Achievement of recommended KDIGO values during the study period.

As summarized in Figure 6, we analyzed the percentage of calcium, phosphorus, and iPTH reaching the recommended range of KDIGO guideline after PTX. Seen from the graph, the majority of patients were able to achieve the KDIGO calcium and phosphate targets, while approximately 34.9% of the patients were within the KDIGO target ranges of iPTH.

### 3.4. Improvement of clinical symptoms (according to the follow-up time)

The clinical symptoms of bone pain and pruritus after operation were significantly improved compared with those before the operation (*p* < 0.05), as shown in Table 4.

**Table 4.** Improvement of clinical symptoms after operation [*n* (%)].

Clinical symptoms	Preop	1.m	*p* value
Bone pain	178 (97.8%)	27 (14.8 %)	<0.001
Pruritus	176 (96.7%）	31 (17.0%)	<0.001

Preop: Preoperative; 1.m: 1 months after operation; categorical data were analyzed with the Chi-square test.

### 3.5. Differences in laboratory indicators and rate of reaching KDIGO recommended range (according to the operative methods)

We analyzed the complete data according to 3 different types of operation, and removed 36 cases of missing patients. Among them, 6 cases underwent S-PTX, 4 cases underwent T-PTX, and 26 cases underwent PTX + AT. After follow-up for 1 year, it was found that the levels of iPTH, Ca, P, and ALP could be significantly reduced by the three surgical methods. The comparison of laboratory indicators among three groups can be seen in Table 5.

**Table 5.** Comparison of the effects of different surgical methods on laboratory indicators.

	All (*n* = 146)	S-PTX (*n* = 28)	T-PTX (*n* = 24)	PTX + AT (*n* = 94)	*p*-value (ANOVA)
iPTH					
Preop	1762.25 (1178.90, 2530.30)	1662.80 (1170.00, 2150.08)	1817.7 (1100.08, 2453.63)	1804.80 (1203.43, 2684.45)	0.533
12.m	60.80 (31.90, 169.40)	253.95 (158.80, 426.80)	41.05 (20.65, 73.23)	57.75 (31.10, 147.23)	0.000
Ca					
Preop	2.72 ± 0.30	2.75 ± 0.24	2.62 ± 0.37	2.74 ± 0.29	0.198
12.m	2.26 ± 0.31	2.43 ± 0.28	2.00 ± 0.14	2.27 ± 0.31	0.000
P					
Preop	2.23 ± 0.49	2.22 ± 0.49	2.14 ± 0.49	2.26 ± 0.49	0.507
12.m	1.50 ± 0.33	1.64 ± 0.40	1.33 ± 0.20	1.49 ± 0.32	0.003
ALP					
Preop	324.50 (161.75, 612.50)	294.50 (130.25, 746.00)	400.00 (126.50, 919.50)	342.50 (173.50, 558.00)	0.529
12.m	99.00 (69.75, 136.75)	101.00 (63.25, 185.00)	125.50 (79.00, 172.75)	92.50 (69.00, 126.00)	0.176

ANOVA: analysis of variance; S-PTX: subtotal parathyroidectomy; T-PTX: total parathyroidectomy; PTX + AT: total parathyroidectomy plus autologous transplantation; iPTH: intact parathyroid hormone; ALP: alkaline phosphatase; Ca: serum calcium; P: serum phosphorus.

Data are presented as the means ± SD or medians and interquartile intervals (25th–75th). A probability value of *p* < 0.001 was considered to be statistically significant.

**Figure 7.**
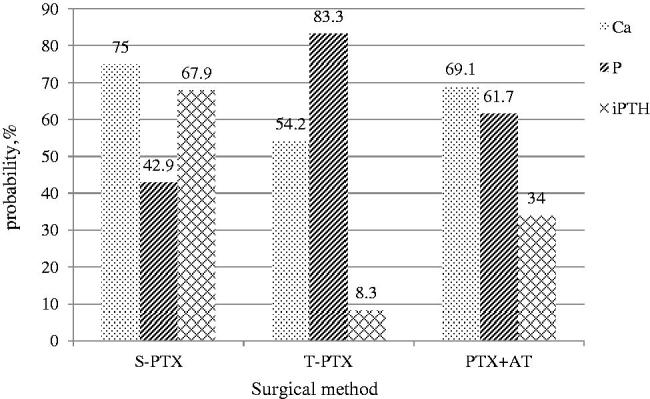
Comparisons of the ratio of each index to the recommended range of KDIGO at 12 months among the S-PTX, T-PTX, and PTX + AT groups.

In addition, according to the type of operation, we made statistics on the ratio of the laboratory indicators of each group reaching the recommended range of KDIGO guidelines after one year of operation. The patients in S-PTX group had the highest ratio of serum calcium and iPTH levels reaching the range recommended by the KDIGO guidelines. T-PTX group had the highest rate of reaching the standard of serum phosphorus level. The rate of reaching the standard of all indexes in PTX + AT group was in the middle as shown in Figure 7.

## 4. Discussion

SHPT disturbs the levels of serum calcium, phosphorus, and ALP. In recent years, large clinical studies, such as the DOPPs and CORES studies, have shown that high or low levels of serum calcium and phosphorus may increase all-cause mortality and cardiovascular mortality in CKD patients and lower their quality of life [22–24]. These findings indicate that it is crucial to control SHPT. The target range of KDIGO guidelines provides a reference for our clinical treatment. However, the control of PTX on the rate of reaching the target range of laboratory indicators in patients with secondary hyperparathyroidism still needs to be further explored.

Similar to a previous study [25], we found that the levels of iPTH, serum calcium, and phosphorus decreased significantly after PTX. In a recent large prospective, multicenter epidemiological observational study in France [26], 9010 incident patients were included. After 6 months of CKD-MBD targeted therapy, the percentage of patients achieving individual KDIGO targets was 58% for intact PTH, 38.7% for phosphate, and 59.9% for calcium. In this study, we found that the percentage of incident dialysis patients with serum levels of calcium within the normal range was similar to that in the Photo-Graphe 3 study. In contrast, the percentage of patients with serum levels of phosphate within the recommended range of KDIGO guidelines was higher than that in their study. This phenomenon suggests that PTX may also improve the disorder of serum calcium and phosphorus metabolism to some extent. In the present study, only 34.9% of the patients reached iPTH values within the optimal KDIGO range at the 12th postoperative months. We considered that this result was related to the fact that PTX caused the concentration of iPTH to be lower than 150 pg/mL in approximately two-thirds of patients, which was very similar to the study by Kovacevic et al. [27] They found that patients undergoing parathyroidectomy achieved the target KDIGO ranges in 11.6–34.9% of patients for calcium and in 53.5–64.7% for phosphate. However, iPTH levels were on target in only 11.6% of patients, with a majority of patients below target. This result was slightly lower than that in this paper, and given the differences in the sample sizes and normal reference ranges of the variables selected by each center, this variability was to be expected. Despite the low achievements of recommended iPTH values after parathyroidectomy, long-term mortality was shown to be lower in patients after surgical treatment compared with dialysis patients receiving only medical treatment [28]. Our study supports that PTX surgery is a safe and effective therapeutic method to reduce the level of iPTH and improve the metabolism of calcium and phosphorus in SHPT that is resistant to medical treatment [29].

It has been reported that bone alkaline phosphatase (BALP) is produced by osteoblasts during the process of bone formation and has been used as a serum marker of bone formation [30]. Unfortunately, our hospital does not routinely perform these examinations. However, in uremic patients, the use of serum total ALP, instead of BALP, to monitor bone metabolism is feasible [31]. Our study found that serum ALP increased significantly at one week after parathyroidectomy, decreased slowly at the third postoperative month, and gradually reached the normal range after 1 year of follow-up. Studies showed BALP levels were negatively correlated with BMD values and the decrease of BALP may contribute to the increase of bone mineral density and the reduction of fracture risk [32,33]. One large cohort study by Maruyama Y et al. [34] demonstrated that higher serum ALP levels were independently associated not only with mortality but also with the incidence of hip fracture in hemodialysis patients. Therefore, strict control of ALP level through PTX has certain clinical significance for increasing bone mineral density, reducing fracture risk, and improving long-term prognosis [35,36].

Pruritus and bone pain, as common clinical symptoms of uremic patients with secondary hyperparathyroidism, not only present considerable burden to patients by seriously reducing their quality of life but also increase the risk of skin ulceration and infection caused by scratching. The pathophysiology of uremic pruritus is not clearly understood. Based on evidence from observational studies, the factors affecting postoperative pruritus seem to be related to disturbance of calcium and phosphorus metabolism [37,38]. Others have found that iPTH was independently associated with the development of severe uremic pruritus [39]. Decreased bone mineral density in patients with secondary hyperparathyroidism is mainly caused by high bone turnover secondary to high levels of circulating PTH [40]. In this process, bone loss is mainly manifested in progressive cortical thinning because of increased bone resorption at the endocortical surface and mineralized bone-like substance formed on the surface of the cortex due to mineralization defects. In severe cases, it is characterized by bone pain and fracture [41,42]. We found that PTX can significantly improve those two clinical symptoms in approximately 1 month after operation, which is closely related to the improvement of calcium and phosphorus metabolism and the reduction of the level of iPTH after parathyroidectomy [43].

The optimal surgical treatment of secondary hyperparathyroidism has not been clearly defined [15]. There have been many studies on the three surgical methods in the past, but the statistics of laboratory indicators reaching the recommended range of KDIGO guidelines are very rare. Consistent with previous study, our study found that all three surgical approaches can control refractory SHPT [44]. As previous studies have shown, S-PTX seems to be able to control SHPT well with fewer complications [45,46]. Our study showed that iPTH and calcium levels reaching the recommended range at a higher rate in the S-PTX group than in the other two groups. However, higher iPTH level also increased the recurrence rate [47]. Although T-PTX can reduce the recurrence rate, the lower level of iPTH may lead to hypoparathyroidism [48]. Some studies recommend PTX + AT as a surgical treatment for SHPT [49,50]. Because of sample size of our research subjects, it is not yet possible to draw the conclusion of which surgical method is the best. But we hope to provide some meager reference for the progress of this research.

However, the present study had some limitations. First, this study was a retrospective study at a single center. Second, the follow-up time was not long enough, and some patients were lost to follow-up. This limitation may have led to some bias in the results, and follow-up efforts need to be further improved. Finally, although the baseline characteristics of our patients were not compared with those of the Global French National Registry (REIN), the data of our center can provide some reference value for clinicians.

In conclusion, the majority of patients who underwent parathyroidectomy were able to reach the range of calcium and phosphorus recommended by the KDIGO guidelines, while approximately 34.9% of the patients reached the range of iPTH. PTX was able to significantly reduce the levels of iPTH and ALP, correct the disorder of calcium and phosphorus metabolism, and improve the clinical manifestations of pruritus and bone pain in uremic patients with SHPT. The three surgical methods have their own advantages and disadvantages, which need to be further explored by multi-center and large sample studies.
